# Assessing Use, Usefulness, and Application of the High Impact Practices in Family Planning Briefs and Strategic Planning Guides

**DOI:** 10.9745/GHSP-D-22-00146

**Published:** 2023-08-28

**Authors:** Maria Carrasco, Saori Ohkubo, Annie Preaux, Christine Galavotti, Alexandria Mickler, Laura Raney, Abdulmumin Saad, Ados Velez May, Heidi Quinn

**Affiliations:** a Office of Population and Reproductive Health, U.S. Agency for International Development/Public Health Institute, Washington, DC, USA.; b Johns Hopkins Center for Communication Programs, Baltimore, MD, USA.; c Tulane University School of Public Health and Tropical Medicine, New Orleans, LA, USA.; d Bill & Melinda Gates Foundation, Seattle, WA, USA.; e FP2030, Washington, DC, USA.; f IBP Network, Washington, DC, USA.; g International Planned Parenthood Federation, Nairobi, Kenya.

## Abstract

**Background:**

In global health, persistent barriers and challenges to bridging the gap between research and practice remain critical to address in most health areas. The High Impact Practices (HIPs) briefs and strategic planning guides aim to bridge the know-do gap in family planning (FP) by facilitating research utilization and knowledge sharing and also providing a summary of experiential knowledge from experts. The purpose of this qualitative study was to assess the use, usefulness, and application of these 2 knowledge products developed by the HIP Partnership for decision-makers and implementers in low- and middle-income countries (LMICs).

**Methods:**

This research used in-depth interviews with FP stakeholders from various LMICs to assess the use, usefulness, and application of 2 HIP products for FP decision-makers and implementers. The analysis was shaped by an adapted logic model framework to assess HIP product reach, engagement, usefulness, learning, and action.

**Results:**

We interviewed 35 FP professionals from January to March 2021. Participants reported that HIP products have a wide reach, have garnered positive engagement, and were useful. Participants generally liked the current format of the HIP products and reported using them to inform program design, guide discussions with partners, enhance personal knowledge, support advocacy work, and strengthen the guidance they provide to colleagues in the field. The participants shared important feedback to improve the development and dissemination of HIP products, particularly a need to enhance local access and use.

**Conclusion:**

The study highlighted the importance of knowledge products, such as the HIP briefs and strategic planning guides, to make evidence and experiential knowledge accessible to a wide audience. These can be valuable tools for policymakers and program implementers to ensure public health practices are evidence-based and integrate experiential knowledge.

## Abstract (precis)

This study highlights the important role that products like the High Impact Practices in Family Planning briefs and strategic planning guides have in increasing accessibility of evidence and experiential knowledge and informing public health practices and programs.

## Teaser

**Key Findings:**

- The High Impact Practices (HIPs) briefs and strategic planning guides can help disseminate knowledge to a wide non-academic audience, support advocacy efforts, and strengthen family planning (FP) programs.
- Interviews with FP professionals revealed a need to enhance local use of HIP briefs and strategic planning guides, adapt or develop additional formats and products for different audiences, and facilitate access to resources to support HIP implementation.

**Key Implications:**

- Creators of knowledge products intended for a non-academic audience, such as decision-makers and program implementers, should consider the importance of using simple, clear language and ensuring quality control of any translated material.
- Requesting input from the main audience of knowledge products is critical to ensure such products address existing gaps and needs, thereby making efficient use of resources.

## INTRODUCTION

A critical challenge in public health is how knowledge and associated practice gaps arise from knowledge translation deficits.^1^ Indeed, it is not enough to produce research; it is also imperative to support its use.^1^ However, bridging the gap between research and practice continues to be a challenge in global health.^2^^,^^3^ This issue was identified, for example, in family planning (FP) research agendas developed in Cote d’Ivoire, Malawi, Mozambique, Nepal, Niger, and Uganda.^4^ Through the process of developing research agendas, stakeholders have identified research and evidence gaps in areas where at least some evidence and research already exists.^4^

Studies have identified several barriers to research utilization, including limited access to relevant and timely research, limited knowledge and skills to critically use research, lack of time, inertia, and cost.^5^^,^^6^ Another important barrier is that most articles published in global health journals are written for a primary audience of scholars and academics and not for practitioners, decision-makers, and policymakers who are situated within specific contexts and must respond to specific local needs when implementing and scaling up programs, practices, and policies.^7^ Furthermore, articles in global health journals emphasize the generalizability of findings over local use, prioritizing the identification of universal truths.^7^

In addition to the challenge of facilitating research utilization, there is an ongoing challenge with systematically capturing experiential learning into global health programs. Normative bodies, such as the World Health Organization (WHO), prioritize the use of high levels of scientific evidence to inform guidance development.^8^ Although this is critical to ensure evidence-based programming, the approach can limit the inclusion of rich qualitative information and less rigorous study designs, as well as experiential learning that must inform public health programs, particularly those where rigorous evidence (such as randomized controlled trials) does not exist or is impractical or not feasible. For example, social and behavior change communication interventions delivered via mass media are often not amenable to being assessed through randomized controlled trials due to methodological challenges.^3^ In fact, WHO commissioned a set of papers to enhance guideline development in recognition of the fact that the original guideline development process was set to develop clinical guidelines, which are very different than the public health, health systems, health promotion, and implementation guidelines that WHO now develops.^9^

Knowledge translation and knowledge management platforms or networks have been important vehicles to facilitate research utilization and experiential knowledge sharing. Such platforms or networks aim to promote research utilization and eliminate gaps between research and practice.^10^ In Malawi, for example, the Malawi Ministry of Health and Dignitas International, a nonprofit organization, formed a partnership to establish the knowledge translation platform KTPMalawi, with the purpose of engaging policymakers, researchers, and implementers on the coordinated generation and use of health sector research. KTPMalawi received some technical support from WHO’s Evidence-Informed Policy Network, which supports the use of health research evidence in low- and middle-income countries (LMICs).^11^ In Nigeria, the Health Policy Committee established in Ebonyi State served as a knowledge translation platform to promote the adoption of evidence-based health policy and interventions.^12^ In the area of FP, the U.S. Agency for International Development (USAID), the Bill & Melinda Gates Foundation (BMGF), and other donors have funded knowledge management activities, such as the Knowledge SUCCESS (Strengthening Use, Capacity, Collaboration, Exchange, Synthesis, and Sharing) project^13^ and The Challenge Initiative (TCI).^14^ Additionally, organizations, such as the Implementing Best Practice (IBP) Network, WHO, FP2030, and others, have been actively working toward the dissemination and uptake of best practices to inform FP programs.

Knowledge translation and knowledge management platforms or networks have been important vehicles to facilitate research utilization and experiential knowledge sharing.

The High Impact Practices (HIPs) in Family Planning Partnership is an important element in this knowledge management ecosystem. The HIP Partnership produces 2 main types of knowledge products: the HIP briefs and the HIP strategic planning guides (SPGs). This qualitative study aimed to assess the use, usefulness, and application of these 2 products developed by the HIP Partnership for decision-makers and implementers in LMICs. Through this study, we hope to improve understanding about the types of knowledge translation products that can support research utilization and knowledge translation in LMICs. This can help to facilitate research utilization and efforts to better synthesize experiential knowledge and evidence from research to share it widely and inform global health programs.

### Background on the HIPs

The HIPs were first created in 2010 after a survey of FP stakeholders revealed little consensus on evidence-based practices in global FP programming. The survey highlighted the need to facilitate knowledge-sharing and consensus-building around what works in FP. At the inception, a small group of leaders in FP worked together to identify a list of practices that if implemented at scale would help countries address unmet need for FP and increase national contraceptive prevalence. Over time, the HIPs evolved to include additional FP-related outcomes, such as delayed marriage, birth spacing, breastfeeding, and, more recently, other outcomes, such as FP service quality and access. Additionally, the process of identifying possible future HIPs is now open to the FP community at large.^15^ The practices are chosen based on scalability, replicability, sustainability, cost effectiveness, potential for application in a wide range of settings, and effectiveness in achieving various FP outcomes.^16^

The groups comprising the HIP Partnership are: (1) the Technical Advisory Group, responsible for the technical oversight of HIP products; (2) a secretariat comprised of 6 organizations providing overall oversight and management and securing funding; (3) the Production and Dissemination team, responsible for the wide dissemination of HIP products; (4) more than 65 partner organizations that provide comments on the HIP products and contribute to dissemination; and (5) the technical expert groups, responsible for updating and developing HIP briefs and SPGs.^17^ The HIP Partnership is structured to engage diverse stakeholders, including researchers, practitioners, advocates, funders, and policymakers.

The HIP briefs and SPGs are written in plain language to ensure the information is accessible to the main audiences of the HIPs, namely, policymakers, program decision-makers (such as donors), and program implementers (including advocates). The 2 types of products are designed to be of short length to distill the essence of the topic covered. The 8-page HIP briefs summarize the evidence from the peer-reviewed and gray literature and provide tips based on experiential knowledge on how best to implement each practice. HIP briefs link programmatic and research considerations by including a set of priority research questions to inform future research.^18^ The HIP SPGs are limited to 4 pages and summarize key considerations to achieve strategic goals and reach programmatic objectives, such as improving equity, engaging men and boys, and implementing FP programs in humanitarian settings.^19^ To ensure high quality, HIP products follow a thorough and participatory technical review process, which includes an internal review by representatives from the cosponsor organizations; community comments by HIP partners and technical experts; and, for the HIP briefs, approval by the HIPs Technical Advisory Group.^20^

The HIP Partnership was first convened by USAID and followed a long history of knowledge dissemination and knowledge management work funded by USAID, starting with *Population Reports*, a journal established in 1973 that became the world’s most widely distributed journal on FP and related health topics until its final issue in 2008.^21^ In the 1980s, USAID began funding projects that focused on knowledge management, particularly on improving the accessibility and use of FP information and services in LMICs. One of the earliest USAID-funded knowledge management projects was the Knowledge for Health (K4Health) project. Launched in 2003, K4Health was a global initiative that worked to improve the quality and accessibility of health information for health workers and policymakers, including those working in FP. The project developed a range of digital tools and platforms, such as the Health Information for All discussion forum; communities of practice focusing on FP topics; and the Toolkits platform, which enabled the creation and hosting of online health information resource libraries.^22^

In 2011, USAID launched the K4Health II project, which focused on building the capacity of health workers and communities to access and use health information, including FP information. The project developed new digital tools and platforms, such as the Mobile for Reproductive Health service, which provided FP information via text messages.

More recently, USAID has continued to support knowledge management for FP through the Knowledge SUCCESS project, which focuses on strengthening the capacity of health workers and organizations to access and use evidence-based information for FP. The Knowledge SUCCESS project has managed various critical HIP Partnership functions to support HIP development and use, including production and dissemination, strategic website management, learning circles, and peer assists.^23^ This body of work reflects a long-standing commitment to improving the availability and accessibility of FP information and services in LMICs.

First convened by USAID, the HIP Partnership has grown to include 5 additional core conveners or sponsoring organizations, including BMGF, International Planned Parenthood Federation, IBP/WHO, United Nations Population Fund, and FP2030, which collaborate to develop the HIP knowledge products and support HIP dissemination and use.

## METHODS

### Design and Sampling

We used purposive sampling to select participants working in the field of FP and reproductive health from both country and global levels. We compiled the list of FP contacts in collaboration with technical advisors from BMGF, FP2030, USAID, and the IBP Network, using the following criteria to prioritize experts from key countries.

- Top 10 countries with the highest number of HIP website users
- Regional representation (e.g., East Africa, West Africa, South Asia, and Latin America)
- Language representation (e.g., English, French, and Spanish)
- Donor country priorities (e.g., BMGF priority FP countries and USAID priority countries for the Office of Population and Reproductive Health)

The initial list of priority countries, which was developed through consensus among the research team, included: Burkina Faso, Burundi, Colombia, Ethiopia, India, Kenya, Mali, Mexico, Nigeria, Pakistan, and Senegal. We aimed to collect data from 30 individuals, composed of 25 country-level participants and 5 participants working across multiple countries (i.e., global-level). We selected the target number based on the research team’s prior experience evaluating knowledge products, which yielded saturation at 25–30 interviews. This is more than the sample size of 9–17 interviews for saturation in qualitative studies, as analyzed in a systematic review of empirically based qualitative studies.^24^ Because we anticipated that most participants would be those who had actively engaged with the HIPs, we contacted individuals from organizations outside of those that participate in the HIP Partnership to identify people who may not have had prior experience using HIP products to learn more about who is not accessing these knowledge products and why and to inform future design and dissemination strategies.

### Data Collection

We invited 65 potential participants via email, asking if they would be willing to participate in an in-depth interview to share their experience with HIP products. Of those invited, 35 individuals replied and agreed to participate. From January to March 2021, 31 interviews were conducted over video conference. Two interviews included 2 participants in the same interview session, for a total of 33 participants. The interviews ranged from 20 to 45 minutes. Two additional participants responded to the interview questions via email, yielding a total of 35 participants. Participants gave verbal consent to participate in the study and have their interviews recorded.

### Analysis

The study team audio-recorded and transcribed the interviews, except for 5 interviews for which the study team took detailed notes or the participants replied to the questions via email. Participant responses in French or Spanish were translated into English. We analyzed the English qualitative data using a combination of manual coding and coding in ATLAS.ti 9. We created the codes and subcodes based on the critical elements of the theoretical framework described in the following section. Also, we developed additional codes as we grouped or segregated the data to identify common themes and unique patterns. Note that some of the authors work in roles as part of the HIP Partnership; however, data collection and analysis were led by authors who do not work directly with the HIP Partnership.

### Theoretical Framework

We used an adapted version of a framework from the Global Health Knowledge Collaborative for this analysis.^25^ Specifically, we created a simplified framework that is specific to HIP processes and retained key elements of the original logic model, including product reach (the breadth of dissemination and distribution of HIP products); engagement (how HIP users spend time and interact with HIP products); usefulness (how practical, applicable, and beneficial the HIP products are perceived to be); and learning and action (outcomes related to awareness, attitudes, intentions, decision-making, practice, and policies) to analyze the data.^25^ These elements are highlighted in our adapted framework in the Figure. Additionally, we also examined participants’ perspectives on the value and benefits of HIP products and their recommendations for improvement. We used each of these areas as themes to organize and present the results in this article.

**FIGURE:**
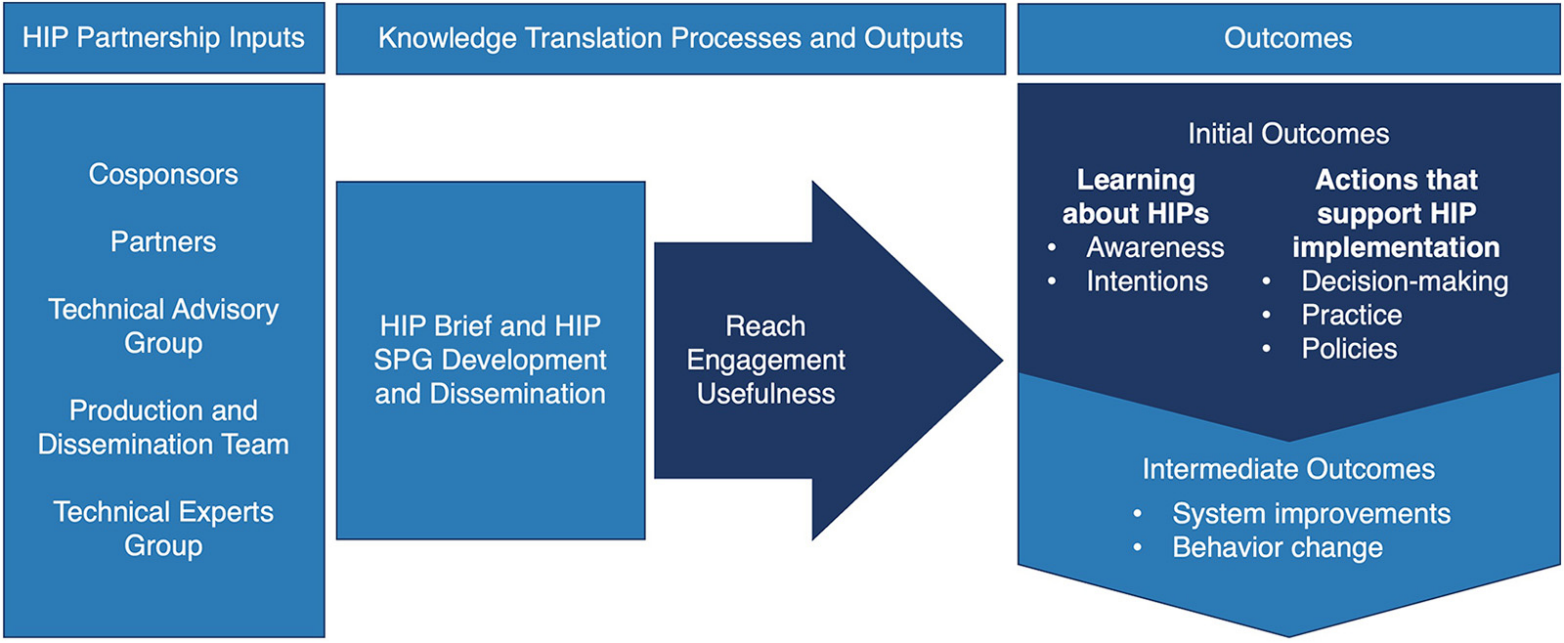
Adapted Framework for HIP Knowledge Translation and Research Utilization^a^ Abbreviations: HIP, High Impact Practice; SPG, strategic planning guide. ^a^The framework used for this analysis was adapted from the Global Health Knowledge Collaborative.^25^ This framework is specific to HIP processes and shows the key aspects of the logic model that were used to guide this analysis: product reach, engagement, usefulness, and learning and action.

### Ethical Approval

Ethical approval for this study was obtained from the Institutional Review Board Office of the Johns Hopkins Bloomberg School of Public Health (IRB No. 15074) on December 15, 2020.

## RESULTS

### Participant Characteristics

Thirty-five FP professionals participated in the evaluation, including 28 country-level participants and 7 global-level participants. Twenty-four interviews were conducted in English, 6 in French, and 5 in Spanish. Approximately two-thirds of participants were female, and participants represented more than 18 organizations, including nonprofits (37%), networks/associations (14%), foundations (11%), national governments (9%), bilateral donors (9%), and multilateral donors (20%).

To facilitate analysis, we divided participant job responsibilities into 2 categories: technical/program officer (71%) or director/senior leader (29%). Participants fell into 3 categories of use: 51% used HIP products and could provide concrete examples of how they were used (“heavy” users); 26% used and read HIP products to increase their knowledge but could not provide concrete examples of product use (“light” users); and 23% did not use HIP products (nonusers).

### Reach, Engagement, and Usefulness

Many country-level participants reported they were introduced to HIP products when attending meetings, workshops, webinars, or trainings. They described global conferences and seminars as important channels for HIP dissemination, and participants valued the ability to pick up printed HIP products. Additionally, many participants mentioned they first heard about HIP products through conversations with coworkers, colleagues, partner organizations, and professional networks (e.g., IBP Network) or when searching for resources on FP project websites. Nearly all participants reported sharing HIP products among colleagues, as well as occasionally with friends or family members. Typically, HIP products were shared electronically by attaching website links and PDFs. Some participants highlighted the importance of sharing the HIPs and putting them in the context of local needs and challenges.

*We are not just sharing [the HIPs] and leaving countries to it. We’re sharing the HIPs as in the context of what is happening in the country by highlighting main points from relevant HIP products.* —Global-level participant working for a professional network

Most participants agreed that the length of the HIP briefs and current format was acceptable.

*The information is very manageable because it’s well summarized; you don’t waste time reading long documents. The information you need is easily obtainable.* —Participant working for an NGO, Burundi

Participants also valued the readability of the briefs and that the briefs used easily understood terminology.

*[HIP products] strike a nice balance, not too dense nor too academic.* —Global-level participant working for an NGO

Participants also found the references sections of products helpful to access more information from original articles and data. They also appreciated the design of the HIP website, noting that “the layout is user friendly, and it is well-done.”

HIP products are translated into 4 languages: English, Spanish, French, and Portuguese. Although participants did not raise concerns with the French or Portuguese translations of the products, a few participants raised issues with Spanish translations of the products, wherein differences in terminology may not align directly with the original English translation. For example, a participant noted that the Spanish translation of HIPs as “proven” and “promising”—categories designated by HIPs based on the strength of evidence—did not denote any difference, as the 2 words used in the translation were very similar.

### Outcomes: Learning and Action

Participants also reflected on which HIP products they recently used, including examples of how the products were used and any related outcomes. Though the majority of participants discussed using HIP briefs, a few participants also spoke about the use of SPGs. Participants described using HIP products to inform program design and strategy, update resources with new data, guide technical or programmatic discussion with partners, and improve personal knowledge.

*We work closely with the Ministry [of Health]. There are always opportunities for referring to materials, so that has been useful. We are currently exploring if there is an opportunity to leverage any of these high-impact practices for strengthening program implementation.* —Participant working for a foundation, India

Participants described using HIP products to inform program design and strategy, update resources with new data, guide technical or programmatic discussion with partners, and improve personal knowledge.

Participants highlighted using multiple HIP products concurrently to align with national FP strategies.

*I receive announcements about HIP products from the HQ colleagues. Some of them are highly relevant, and I get ideas from HIP products relevant to the country’s activities. For example, the HIP brief on community health workers relates to health extension workers in Ethiopia.* —Participant working for a bilateral donor agency, Ethiopia

HIP products are also used by participants for advocacy, capacity building, and other country support work. Products were cited as useful resources for establishing FP2020 Country Commitments, contextualizing webinars in specific settings, and bringing practices to scale. Some participants also mentioned how HIP resources could be adapted to address challenges posed by the COVID-19 pandemic. For example, a participant reflected on using and adapting the Pharmacies and Drug Shops Brief.

*When the pandemic started, we were able to repurpose their pharmacy strategy to respond to COVID-19. The information was easily transferable. We were able to add COVID information. This quick, easy transition was possible because the HIP was well-documented in advance, facilitating the adaptation.* —Participant working for an NGO, Mexico

Participants also noted how using the HIPs may have contributed to other FP programming outcomes. Many participants commented that knowledge gained from HIP products translated into improved practice implementation and strengthened guidance provided to field-based colleagues. A participant mentioned the changes observed from using the adolescent SPG.

*One specific example that comes to mind is opening community discussions with youth about sexual and reproductive health. Young adults did not feel comfortable addressing [sexual and reproductive health] issues either with their parents or community leaders. We implemented community talks that involved youth, parents, community leaders, and health providers. We noticed that providers also were not trained in providing youth-friendly services. It was very successful because now we have noticed that more youth are able to talk about their needs for family planning.* —Participant working for an NGO, Togo

Although participants found the products to be useful, participants felt that it is difficult to understand the direct contributions of HIP products to FP program outcomes because HIP products are frequently used in conjunction with other resources. Thus, HIP products contribute to the larger context of FP guidelines, tools, and resources.

*In terms of HIPs products themselves, they could only be contributory because some of those ideas may not be new, just be presented in a way that’s easier to understand and maybe gives focus because they’re short. I would not go as far as being an attributor to FP programs because we do have many people with significant technical expertise.* —Participant working for a multilateral donor agency, Nigeria

Around one-quarter of participants indicated they had not used any HIP products in their work, despite their familiarity with the HIPs. Some participants indicated that issues covered by HIPs were well understood among their FP colleagues, and thus, the content was too basic for countries that have worked in FP programs for an extended period of time.

*We have tried many things covered by HIP products. Family planning is an advanced science and practice here. HIP products are for the global audience, where these things are relatively new.* —Participant working for a foundation, India

Nearly all participants who were not familiar with the HIPs indicated that the products seemed very interesting and relevant to their work and expressed their intention to begin using and sharing HIP products.

*I oversee all the member organizations that work in the field of family planning. These organizations develop and implement projects and would be the ones using these products. In my capacity, I have not come across any of these products. Now that I have seen the HIP products website, I will go and see how I can use these resources to train others in the coalition.* —Participant working for a professional network, Mali

### Value and Benefit of HIP Products

Participants widely cited HIP products as helpful resources to inform FP programs. The results indicate that several HIP users consider HIP products a global good—easy to access and available to all to use as needed to support FP programming. Many participants discussed how HIP products have helped them to promote and reinforce some practices that were considered or were already in use, serving both information-sharing and advocacy purposes. Many participants mentioned using HIP products to inform their partners, counterparts, and other organizations about HIPs.

*It’s good to refer to [HIPs] in the conversations in informing, engaging, and perhaps even convincing the government, internal team, [and] partners whom you are trying to influence… [HIP products are] effective in bolstering the argument.* —Participant working for a foundation, India

Many participants discussed how HIP products have helped them to promote and reinforce some practices that were considered or were already in use, serving both information-sharing and advocacy purposes.

Participants found it helpful to review HIP products before beginning a program, project, or activity to ensure all essential elements of a particular practice are included. Further, HIP products help stakeholders determine which practices are relevant and feasible for specific contexts.

*Make sure to include every element that HIP offers so that you use the experience of others; you do not need to reinvent the wheel. You can just go ahead and deliver the things that do work.* —Participant working for a Latin American regional organization

*It’s really important, when providing technical oversight to the government, to say, “This is what we need to do, these are the areas where we are weak, and these are opportunities for us.” When developing strategic plans and concept notes, I need to have the information on what’s working elsewhere and determine what will work in our context.* —Participant working for a multilateral donor agency, Nigeria

### Recommendations From Study Participants

Participants elaborated on the importance of extending dissemination efforts beyond higher-level stakeholders (e.g., governments, policymakers, and implementers) to the community level.

*We need a dissemination strategy so that HIP products are going beyond the national capital and reaching community-based organizations.* —Participant working for a foundation, India

*We need to map grassroots organizations working on family planning and reach out to them to join a global network like IBP. Then, they can easily access HIP products and other updates.* —Participant working for a bilateral donor, India

Participants also discussed the importance of streamlining the dissemination of HIP products within national structures, such as technical working groups and other platforms where ministries of health, donors, and implementing partners convene. Participants mentioned using the following additional channels to strengthen local dissemination.

- Basic or refresher training and seminars by the government (to incorporate HIPs into the national curriculum), implementing organizations, or civil societies to target the right audiences, including community health workers, midwives, and other service providers.
- Communities of practice and virtual discussions on HIP products at the country level.
- Targeted in-person outreach and on-site dissemination for local-level FP practitioners.
- Direct email announcements to sexual health associations, nurse associations, and other relevant professional networks.
- More frequent webinars, equipped with simultaneous translation, to reach new staff.

Participants also suggested adapting HIP content into other formats. For example, participants suggested making the list of organizations, project examples, country comparisons, and regionally relevant experiences more prominent on the website and in HIP products and (re)introducing tools to showcase and monitor where HIPs are implemented to replicate successful initiatives.

*I remember we used to have a map on the website showing countries implementing HIPs…If not a visual map, to monitor HIP products use, we could use a proxy indicator, such as the number of downloaded documents from countries… We know 1,000 people downloaded documents, [but that] does not mean that the government is implementing HIPs. At least, the number of downloaded documents being publicly available could be a good source of information for policymakers…It will facilitate learning by the governments across the region.* —Global-level participant working for a multilateral donor agency

Participants also expressed the need to engage a wider audience, including implementing partners directly involved in service delivery and capacity-strengthening of health care providers and community volunteers.

*When developing HIP products, we could consider engaging larger audiences beyond the technical working groups representing regions as we now have the advantage of meeting virtually.* —Participant working for a bilateral donor agency, India

Participants made the following suggestions to consider different formats or auxiliary products to meet the needs of different audiences.

- Create shorter documents for decision-makers who need to know the key points
- Simplify documents (make them less technical) for people working in the communities
- Develop additional sections, resources, tools, and models for those needing advanced guidance on HIPs
- Place the key messages prominently at the top of the page to capture readers’ attention
- Adapt content to the local needs of each region and country
- Establish a forum on the website where people connect with organizations doing similar work and exchange ideas, activities, and experiences
- Add a document search function on the website
- Showcase HIP content in other formats, such as short videos, animated videos, audio recordings, presentations/slides, infographics, and other audiovisual materials

## DISCUSSION

### Reach, Engagement, and Usefulness

Participants indicated that they perceived the 2 types of HIP products to have a wide reach and have garnered positive engagement. The participants who currently access and use these 2 HIP products described the products as useful for their work. The majority of respondents had heard of the HIPs, and most had used the HIP products. This qualitative finding triangulates the quantitative data from the HIP website, which points to a large number of users (101,365 unique users from October 2020 to September 2021).

However, a few respondents had not heard about the HIPs, which suggests a need for enhanced dissemination. It is likely that this is particularly needed among local organizations that lack connections with global organizations and actors, particularly the organizations comprising the HIP Partnership. This finding points to the difficulty of ensuring a wide reach of knowledge products developed for a wide audience across LMICs. A review of knowledge dissemination interventions in health research found 3 main forms of communication used by such interventions: written materials, electronic materials, and interpersonal communication activities or events.^26^ The review also identified the importance of disseminating to specific audiences, such as people in clinical settings or end users of the knowledge that is disseminated.^26^

To enhance knowledge product dissemination, knowledge-sharing platforms in global health have formed linkages with other knowledge translation platforms and regional organizations. For example, the Zambia Forum for Health Research has benefited from efforts to create demand for research evidence among policymakers and communities and to invest in network-building, including creating alliances with other knowledge translation platforms in Africa.^27^ Linkages between local knowledge translation and knowledge-sharing platforms may also enable more variety in the mode of disseminating information, such as round-table discussions, presentations, research digests, interactive workshops, and tailored messages to mobile devices.^28^^,^^29^

The results also suggest that the 2 types of HIP products are helping to address an important need among policymakers, decision-makers, program implementers, and advocates in the FP community for accessible, practical, and useful information to support the design and implementation of evidence-based FP policies and programs. In the global health space, this finding corroborates the need among non-academic cadres of professionals for evidence that is easy to digest and access, as well as the need to share experiential knowledge in a systematic way.

The results suggest that the HIP briefs and SPGs help to address an important need for accessible, practical, and useful information to support the design and implementation of evidence-based FP policies and programs.

A few participants had concerns that the information in HIP products was too basic or general to guide FP professionals in specific contexts they considered as “advanced.” In such cases, it would be important to consider segmenting the target audience and offering knowledge products and other resources based on different levels of expertise or the level of detail desired and needed by various target audiences.

The finding related to translation from English to Spanish highlights the need to implement translation quality control and pretesting strategies that ensure concepts and nuances are not lost in translation. Concerns about language have been identified in other evaluations of knowledge-sharing strategies^30^; language and cultural adaptation of knowledge products are crucial considerations and must be adequately resourced to be done well.

### Learning and Action Outcomes

Additional evaluation is needed to assess the impact of HIP products on FP-related outcomes, but this study has suggested that the 2 HIP products assessed can contribute to advancing research utilization and experiential knowledge sharing and can have a positive influence in strengthening global health programs related to FP. Through participants’ responses, we found examples of knowledge use in practice that was facilitated by the use of HIPs products. About 75% of participants used HIP products for decision-making purposes to inform policy, strategy, and practice. These participants represented a wide range of professionals—including policymakers, technical advisors, program officers, and network coordinators—from various sectors, including government agencies, donor agencies, professional associations, and nonprofit organizations. Many participants shared HIP products with others in their networks and used the products in tandem with other resources to support program implementation. Although, in general, HIP products were found to be useful, participants also indicated an opportunity for the HIP Partnership to do more to facilitate technical exchange and provide additional resources, tools, and models to those who need advanced guidance to implement HIPs. This suggests an opportunity to support learning and action by facilitating access to additional related resources. There are some interesting examples of how other knowledge translation and knowledge-sharing platforms support research utilization for implementation. For example, in addition to producing evidence briefs, 2 knowledge translation platforms on health system policymaking in Cameroon and Uganda also conduct stakeholder analyses and priority-setting exercises, engage stakeholders in dialogue related to evidence, and offer “rapid response” services to answer urgent requests for evidence.^31^ A knowledge translation platform in Nigeria hosted a stakeholders’ policy dialogue to discuss the policy briefs developed by the platform and their application.^12^ A knowledge translation platform in Brazil offered capacity-building activities, including courses and workshops to develop, assess, and use evidence briefs.^32^ The HIP Partnership could easily support the implementation of the practices summarized in the HIP briefs by enhancing website linkages with existing platforms built to facilitate FP technical exchanges, such as the IBP Network,^33^ FP insight,^34^ The Challenge Initiative,^35^ and conferences including the International Conference on Family Planning.^36^ The role of knowledge-sharing platforms in supporting program implementation and scale-up should be further explored.

### Strengths and Limitations

One strength of this study is that HIP product users and nonusers were engaged in providing recommendations. Engaging end users in providing feedback is just a small step toward the HIP Partnership’s efforts to ensure the participation of stakeholders from LMICs and increase inclusivity by engaging a diverse set of actors and voices. The richness of recommendations from study participants highlights that requesting input from the main audience of knowledge products is critical to ensure such products address existing gaps and needs, thereby making efficient use of resources. Additionally, this input can also help to uncover gaps and needs not yet addressed, help to facilitate research utilization, and improve knowledge-sharing to strengthen programs.

A limitation of this study is that the purposive sampling technique and small sample size used for in-depth interviews do not allow us to generalize these findings to the broader community of FP stakeholders around the world. Feedback on the usefulness and use of HIP products may vary in other countries and regions that were not represented in this study. As we have noted, we may also be missing the voice of FP stakeholders who work at a more local level. As sampling was based on HIP cosponsor priority countries and countries that frequently access the HIP website, these participants may have been more likely to have heard of the HIP Partnership and be using HIP products than the overall global FP community. Finally, it is important to note the relatively small sample size of 35 study participants representing a broad range of geographic regions does not allow for broader conclusions related to the reach of HIP products.

## CONCLUSION

This study found widespread usage of 2 HIP products among participants, and the results suggest a need to enhance local use and facilitate access to resources to support HIP implementation. The results also highlight the importance of making available knowledge products that are written for a non-academic audience, using simple and clear language. The findings highlight the utility of knowledge translation and knowledge-sharing efforts to enhance global health programs. Continued evaluation of the HIP Partnership’s knowledge translation processes will be crucial to generating more information about what is most relevant and useful to facilitate research utilization and knowledge translation while being more responsive to stakeholders in LMIC contexts.

## Notes

**Peer Reviewed**

**First published online**: July 5, 2023.
