# Corrigendum: Carrasco M et al., Assessing Use, Usefulness, and Application of the High Impact Practices in Family Planning Briefs and Strategic Planning Guides

**DOI:** 10.9745/GHSP-D-23-00445

**Published:** 2023-12-22

See corrected article .

The article “Assessing Use, Usefulness, and Application of the High Impact Practices in Family Planning Briefs and Strategic Planning Guides” by Maria Carrasco et al. (Volume 11, Issue 4) contained incorrect information on when the Knowledge for Health project operated and the digital tools and platforms that the project developed and supported.

The article has been corrected accordingly.

